# Prognostic Correlations between ABO Blood Group and Pre-Treatment Plasma Epstein-Barr Virus DNA in Patients with Nasopharyngeal Carcinoma Receiving Intensity-Modulated Radiotherapy

**DOI:** 10.1371/journal.pone.0166194

**Published:** 2016-11-11

**Authors:** Hao Peng, Lei Chen, Wen-Fei Li, Yuan Zhang, Li-Zhi Liu, Li Tian, Ai-Hua Lin, Ying Sun, Jun Ma

**Affiliations:** 1 Department of Radiation Oncology, Sun Yat-sen University Cancer Center, State Key Laboratory of Oncology in Southern China, Collaborative Innovation Center for Cancer Medicine, Guangzhou, 510060, Guangdong Province, People’s Republic of China; 2 Imaging Diagnosis and Interventional Center, Sun Yat-sen University Cancer Center, State Key Laboratory of Oncology in Southern China, Collaborative Innovation Center for Cancer Medicine, Guangzhou, 510060, Guangdong Province, People’s Republic of China; 3 Department of Medical Statistics and Epidemiology, School of Public Health, Sun Yat-sen University, Guangzhou, 510080, Guangdong Province, People’s Republic of China; Southern Illinois University School of Medicine, UNITED STATES

## Abstract

**Purpose:**

The objective of this study is to assess the prognostic value of ABO blood group in nasopharyngeal carcinoma (NPC) treated by intensity-modulated radiotherapy (IMRT).

**Patients and Methods:**

We retrospectively reviewed the data on 1397 patients with non-metastatic, newly diagnosed NPC treated using IMRT. Patient survival between different ABO blood groups were compared using log-rank test. Cox hazards model was adopted to establish independent prognostic factors.

**Results:**

In our study, the distribution of the A, B, AB and O blood groups was 26.6% (372/1397), 26.2% (366/1397), 5.2% (73/1397) and 42.0% (586/1397), respectively. The cut-off value of pre-treatment Epstein-Barr virus (EBV) DNA based on disease-free survival (DFS) was 1355 copies/ml (area under curve [AUC], 0.649; sensitivity, 0.76; specificity, 0.496) for the whole cohort. Estimated four-year DFS, overall survival (OS), distant metastasis-free survival (DMFS) and locoregional relapse-free survival (LRRFS) rates were 81.7%, 89.2%, 89.4% and 92.3% for blood group A; 82.1%, 89.3%, 89.0% and 92.0% for group B; 83.3%, 88.1%, 86.2% and 95.5% for group AB, 80.9%, 90.7%, 88.4% and 90.2% for group O (*P* > 0.05 for all rates). Multivariate analysis revealed ABO blood group was not an independent prognostic factor for DFS, OS, DMFS or LRRFS (*P* > 0.05 for all rates) after adjusting for plasma EBV DNA in either the whole cohort or subgroup analysis by gender.

**Conclusions:**

The prognostic value of ABO blood group may be limited for patients with NPC in the era of IMRT, and no substantial correlation between ABO blood group and plasma EBV DNA was observed.

## Introduction

Nasopharyngeal carcinoma (NPC), which arises from the nasopharyngeal epithelium, has an extremely unbalanced global distribution [1]. Worldwide, 86,500 cases of NPC were reported in 2012, with 71% of all new cases in east and Southeast Asia, including southern China [1]. Due to anatomic constraints and its high degree of radiosensitivity, radiotherapy is the main treatment for non-metastatic NPC. The TNM staging system is the most reliable method for guiding clinical treatment and predicting prognosis [2]. The prognosis of early-stage NPC is satisfactory; however, locoregionally-advanced disease still has a poor prognosis; the 5-year overall survival rates are 87–96% for stage I-II and 67–77% for stage III-IV [3]. Therefore, additional novel prognostic factors need to be identified to select patients at high risk and devise individual treatment strategies.

Relationship between the ABO blood group and prognosis have been reported in many tumor types, including breast cancer [4], pancreatic cancer [5, 6], esophageal squamous cell carcinoma [7, 8], laryngeal squamous cell carcinoma [9] and non-small cell lung cancer [10]. Seemingly, the ABO blood group may be a useful prognostic factor and it also plays an important role in NPC. However, outcomes from previous studies addressing this issue remain controversial [11–13] and therefore needs to be further investigated.

NPC has been proven to be an Epstein-Barr virus (EBV)-associated cancer [14, 15]. Over the last decade, plasma EBV DNA has been proven to be the most successful biomarker for detecting and predicting prognosis in NPC [16–20]. Moreover, Zhang et al. [13] demonstrated that male patients with stage II/III NPC, blood group A and high immunoglobulin A antibodies to EBV viral capsid antigen (VCA-IgA) level had a favorable prognosis. Therefore, it is reasonable to hypothesize that a potential prognostic relationship may exist between the ABO blood group and the pre-treatment EBV DNA (pre-DNA) level in NPC. However, no relevant work has been performed to confirm this relationship. Thus, on the basis of this premise, we conducted this retrospective study to explore the prognostic value of the ABO blood group and assess the potential relationship between the ABO blood group and pre-DNA in patients with NPC undergoing intensity-modulated radiotherapy (IMRT).

## Materials and Methods

### Patient Selection

We retrospectively analyzed data from 1811 patients with newly biopsy-confirmed NPC with no evidence of distant metastasis treated between November 2009 and February 2012 at Sun Yat-sen University Cancer Center. Eligibility criteria were: (1) World Health Organization pathological type II or III; (2) pre-DNA data; (3) blood group data; (4) no previous other malignant cancers. In total, 1397 (77.1%) patients were eligible for this study. The protocol and procedures employed in this study were reviewed and approved by the Research Ethics Committee of Sun Yat-sen University Cancer Center. Informed consent was obtained from all patients before treatment.

### Clinical Staging

Routine staging included a complete medical history and clinical examination of head and neck, direct fiber-optic nasopharyngoscopy, magnetic resonance imaging (MRI) of skull base and entire neck, chest radiography, a whole-body bone scan, abdominal sonography, and positron emission tomography (PET)-CT if indicated.

All patients had a dental evaluation before radiotherapy and were restaged according to the 7^th^ edition of the International Union against Cancer/American Joint Committee on Cancer (UICC/AJCC) staging system [21]. All MRI materials and clinical records were reviewed to minimize heterogeneity in restaging. Two radiologists employed at our hospital separately evaluated all scans; disagreements were resolved by consensus.

### Real-Time Quantitative EBV DNA PCR

Pre-DNA was measured before treatment using real-time quantitative PCR, as previously described [22], using an assay developed for detection of plasma EBV DNA that targets the *BamH*I-W region of the EBV genome (primers: 5`-GCCAGAGGTAAGTGGACTTT-3`and 5`-TACCACCTCCTCTTCTTGCT-3`; dual fluorescence-labeled oligomer probe: 5`-(FAM)CACACCCAGGCACACACTACACAT(TAMRA)-3`). EBV genome sequence data were obtained from the GenBank sequence database.

### Treatment

All patients were treated with definitive IMRT at Sun Yat-sen University Cancer Center. A high-resolution planning computed tomography scan with contrast was taken from the vertex down to 2 cm below the sternoclavicular joint (slice-thickness, 3 mm). Prescribed doses were 66–72Gy at 2.12–2.43Gy/fraction to the planning target volume (PTV) of the primary gross tumor volume (GTVnx), 64–70Gy to the PTV of the GTV of involved lymph nodes (GTVnd), 60–63Gy to the PTV of high-risk clinical target volume (CTV1), and 54–56Gy to the PTV of low-risk clinical target volume (CTV2). Before treatment, we recommended radiotherapy alone for stage I, concurrent chemoradiotherapy for stage II, and concurrent chemoradiotherapy (CCRT) +/- neoadjuvant (NCT)/adjuvant chemotherapy (ACT) for stage III to IVA-B according to institutional guidelines. Neoadjuvant or adjuvant chemotherapy consisted of cisplatin with 5-fluorouracil (PF) or cisplatin with docetaxel (TP) administered every three weeks for two or three cycles. Concurrent chemotherapy consisted of cisplatin given weekly or on weeks 1, 4 and 7 of radiotherapy.

### Follow-Up and Statistical Analysis

Patient follow-up was measured from first day of therapy to day of last examination or death. Patients were examined at least every three months during first two years, with follow-up examinations every six months thereafter until death. End-points (time to first defining event) included disease-free survival (DFS), overall survival (OS), distant metastasis-free survival (DMFS) and locoregional relapse-free survival (LRRFS). Receiver operating curve (ROC) analysis was used to identify the cut-off value for pre-DNA based on DFS. The Chi-square test was used to compare clinical characteristics. Life-table estimation was performed using the Kaplan-Meier method; differences were compared using the log-rank test. The multivariate Cox proportional hazards model was used to estimate hazard ratios (HR) and 95% confidence intervals (CI). All statistical tests were two-sided; *P* < 0.05 was considered statistically significant. STATA statistical package (STATA 12; StataCorp LP, College Station, Texas, USA) was used for all analyses.

## Results

### Pre-DNA Cut-Off Value

Median pre-DNA was 2190 copies/ml (interquartile range, 0–17300) for the entire cohort, 2840 copies/ml (interquartile range, 0–24925) for patients with blood group A, 1585 copies/ml (interquartile range, 0–12600) for blood group B, 1370 copies/ml (interquartile range, 0–12000) for blood group AB and 2830 copies/ml (interquartile range, 0–15875) for blood group O. According to the ROC curve analysis based on DFS, the optimal pre-DNA cut-off value was 1355 copies/ml (area under curve [AUC], 0.649; sensitivity, 0.76; specificity, 0.496) for the whole cohort. Patients with pre-DNA ≥ 1355 copies/ml were classified into the pre-H group and patients with pre-DNA < 1355 copies/ml into the pre-L group.

### Baseline Characteristics

The clinical characteristics of the 1397 patients are summarized in Table 1. The proportion of patients with blood groups A, B, AB and O were 26.6% (372/1397), 26.2% (366/1397), 5.2% (73/1397) and 42.0% (586/1397), respectively. Median age for the whole cohort was 45 years (range, 14–78 years). The balance of clinicopathological features was only compared between patients with blood group A and patients with non-A blood groups; no significant differences were observed between these two groups with regard to any tumor or host factor (*P* ≥ 0.05 for all rates).

**Table 1:** Baseline characteristics of the 1397 patients with nasopharyngeal carcinoma.

Characteristic	Blood group					*P*^*a*^
	A	B	AB	O	non-A	
	(n = 372)	(n = 366)	(n = 73)	(n = 586)	(n = 1025)	
	(%)	(%)	(%)	(%)	(%)	
Age (years)						0.361
< 45	173 (46.5)	183 (50.0)	27 (37.0)	295 (50.3)	505 (49.3)	
≥ 45	199 (53.5)	183 (50.0)	46 (63.0)	291 (49.7)	520 (50.7)	
Gender						0.106
Male	270 (72.6)	279 (76.2)	54 (74.0)	454 (77.5)	787 (76.8)	
Female	102 (27.4)	87 (23.8)	19 (26.0)	132 (22.5)	238 (23.2)	
Family history						0.898
Yes	104 (28.0)	108 (29.5)	22 (30.1)	153 (23.1)	283 (27.6)	
No	268 (72.0)	258 (70.5)	51 (69.9)	433 (73.9)	742 (72.4)	
Smoking						0.623
Yes	144 (38.7)	135 (36.9)	31 (42.5)	216 (36.9)	382 (37.3)	
No	228 (61.3)	231 (63.1)	42 (57.5)	370 (63.1)	643 (62.7)	
Drinking						0.072
Yes	37 (9.9)	48 (13.1)	12 (16.4)	79 (13.5)	139 (13.6)	
No	335 (90.1)	318 (86.9)	61 (83.6)	507 (86.5)	886 (86.4)	
T category ^b^						0.294
T1-2	132 (35.5)	126 (34.4)	22 (30.1)	185 (31.6)	333 (32.5)	
T3-4	240 (64.5)	240 (65.6)	51 (69.9)	401 (68.4)	692 (67.5)	
N category ^b^						0.486
N0-1	273 (73.4)	282 (77.0)	55 (75.3)	434 (74.1)	771 (75.2)	
N2-3	99 (26.6)	84 (23.0)	18 (24.7)	152 (25.9)	254 (24.8)	
Overall stage^b^						0.279
I	15 (4.0)	20 (5.5)	4 (5.5)	29 (4.9)	53 (5.1)	
II	83 (22.3)	80 (21.8)	15 (20.6)	117 (20.0)	212 (20.7)	
III	163 (43.8)	171 (46.7)	35 (47.9)	289 (49.3)	495 (48.3)	
IVA-B	111 (29.9)	95 (26.0)	19 (26.0)	151 (25.8)	265 (25.9)	
Pre-DNA (copies/ml)						0.568
< 1355	162 (43.5)	175 (47.8)	36 (49.3)	253 (43.2)	464 (45.3)	
≥ 1355	210 (56.5)	191 (52.2)	37 (50.7)	333 (56.8)	561 (54.7)	
Chemotherapy						0.663
Yes	327 (87.9)	319 (87.2)	63 (86.3)	510 (87.0)	892 (87.0)	
No	45 (12.1)	47 (12.8)	10 (13.7)	76 (13.0)	133 (13.0)	

Abbreviation: pre-DNA = pre-treatment Epstein-Barr virus DNA

^a^ Calculated using the Chi-square test or Fisher’s exact test if indicated.

^b^ 7^th^ edition of the AJCC/UICC staging system.

### Failure Patterns

Up to the last day of follow-up (August 2015), 149 (10.7%) patients died and 87 (6.2%) patients were lost to follow-up. The median follow-up duration was 49.7 (range, 1.3–76.4) months for the entire cohort and 50.3 (range, 1.3–76.4) months for the survival patients. Treatment failure patterns for the whole cohort stratified by blood group are listed in Table 2. Obviously, distant metastasis has been the main failure pattern. No significant difference was observed between the blood A group and non-A group with regard to local, regional and distant failure patterns (P > 0.05 for all rates).

**Table 2:** Treatment failure patterns for the 1397 patients with nasopharyngeal carcinoma.

Failure patterns	Blood group					*P*^a^
	A	B	AB	O	Non-A	
	(*n =* 372)	(*n =* 366)	(*n =* 73)	(*n =* 586)	(*n =* 1025)	
	(%)	(%)	(%)	(%)	(%)	
Local only	18 (4.8)	16 (4.4)	3 (4.1)	33 (5.6)	52 (5.1)	0.859
Regional	17 (4.6)	18 (4.9)	1 (1.4)	26 (4.4)	45 (4.4)	0.885
Total locoregional	30 (8.1)	29 (7.9)	4 (5.5)	55 (9.4)	88 (8.6)	0.757
Distant	39 (10.5)	39 (10.7)	10 (13.7)	68 (11.6)	117 (11.4)	0.625
Death	43 (11.6)	43 (11.7)	9 (12.3)	54 (9.2)	106 (10.3)	0.515

^a^ Calculated using the Chi-square test or Fisher’s exact test if indicated.

### Prognostic Impact of ABO Blood Group

**Fig 1:**
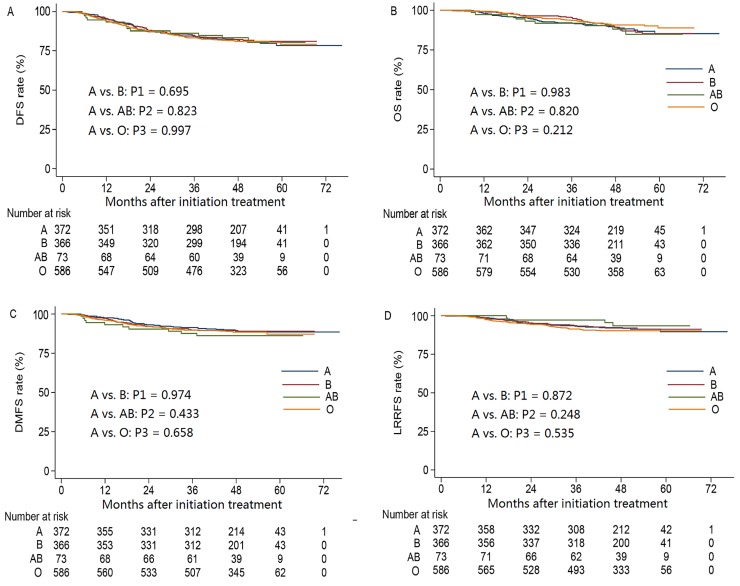
Kaplan-Meier curve analysis for patients with NPC stratified by different blood groups. (A) Disease-free survival curves. (B) Overall survival curves. (C) Distant metastasis-free survival curves. (D) Locoregional relapse-free survival curves.

**Fig 2:**
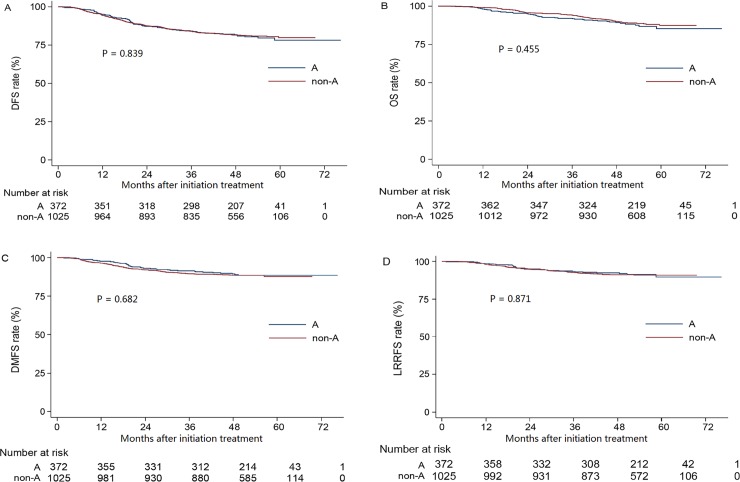
Kaplan-Meier curve analysis for patients with NPC stratified by A and non-A blood groups. (A) Disease-free survival curves. (B) Overall survival curves. (C) Distant metastasis-free survival curves. (D) Locoregional relapse-free survival curves.)

The 4-year DFS, OS, DMFS and LRFFS rates for the entire cohort were 81.6%, 89.8%, 88.7% and 91.5%, respectively. The univariate analysis results was listed in Table 3. Compared to patients with blood group A (control group), blood group B, AB and O were not associated with significant survival benefits in terms of 4-year DFS, OS, DMFS and LRFFS (Fig 1). Additionally, when patients with blood group A were compared to the patients with non-A blood groups, no significant differences in 4-year DFS, OS, DMFS and LRFFS were observed (Fig 2).

**Table 3:** Univariate analysis of the 1397 patients with nasopharyngeal carcinoma.

Blood group	4-year Survival outcomes			
	DFS	OS	DMFS	LRRFS
	(%)	(%)	(%)	(%)
A (*n =* 372)	81.7	89.2	89.4	92.3
B (*n =* 366)	82.1	89.3	89.0	92.0
AB (*n =* 73)	83.3	88.1	86.2	95.5
O (*n =* 586)	80.9	90.7	88.4	90.2
Non-A (*n =* 1025)	81.5	90.0	88.5	91.2
*P*1 (A vs. B)	0.695	0.983	0.974	0.872
*P*2 (A vs. AB)	0.823	0.820	0.433	0.248
*P*3 (A vs. O)	0.997	0.212	0.658	0.535
*P*4 (A vs. non-A)	0.839	0.455	0.682	0.871

Abbreviation: DFS = disease-free survival; OS = overall survival; DMFS = distant metastasis-free survival; LRRFS = locoregional relapse-free survival.

Multivariate analysis was performed to adjust for various prognostic factors (Table 4). Consistent with the univariate analysis, the individual blood groups had no significant prognostic value. In further multivariate analysis assessment of prognostic value, blood group (A vs. non-A) had no prognostic value for DFS (HR, 0.999; 95% CI, 0.760–1.314; *P* = 0.996), OS (HR, 0.866; 95% CI, 0.607–1.236; *P* = 0.428), DMFS (HR, 1.109; 95% CI, 0.771–1.595; *P* = 0.577) or LRFFS (HR, 1.100; 95% CI, 0.726–1.667; *P* = 0.654).

**Table 4:** Multivariate analysis outcomes for the 1397 patients with nasopharyngeal cancer.

Endpoints	Variable	*P*^a^	HR	95% CI for HR
DFS	Age	0.035	1.302	1.019–1.664
	Pre-DNA	< 0.001	2.464	1.843–3.296
	N category ^b^	< 0.001	1.805	1.401–2.327
OS	Age	0.001	1.792	1.279–2.510
	Pre-DNA	< 0.001	2.257	1.503–3.389
	T category ^b^	0.002	1.983	1.292–3.043
	N category ^b^	< 0.001	2.282	1.639–3.177
DMFS	Pre-DNA	< 0.001	3.538	2.300–5.441
	N category ^b^	< 0.001	2.363	1.714–3.258
LRRFS	Pre-DNA	0.001	1.965	1.327–2.909

Abbreviations: DFS = disease-free survival; OS = overall survival; DMFS = distant metastasis-free survival; LRRFS = locoregional relapse-free survival; Pre-DNA = pre-treatment Epstein-Barr virus DNA; HR = hazards ratio; CI = confidence interval.

^a^ Calculated using an adjusted Cox proportional hazards model.

^b^ 7^th^ edition of the AJCC/UICC staging system.

### Subgroup Analysis Based on Pre-DNA and Gender

To further address the relationship between pre-DNA and blood group, subgroup analysis was conducted based on pre-DNA and gender (Table 5). In subgroup analysis by pre-L or pre-H, no significant differences of 4-year DFS, OS, DMFS or LRRFS were observed in male or female patients with blood group B, AB and O versus blood group A (*P* > 0.05 for all rates). Furthermore, no significant differences were observed in subgroup analysis of patients with blood group A and patients with non-A blood groups (*P* > 0.05 for all rates). Multivariate analysis confirmed blood group was not an independent prognostic factor for any end-point.

**Table 5:** Subgroup analysis based on pre-DNA and gender for the 1397 patients with nasopharyngeal cancer.

Blood group	Pre-L								Pre-H							
	Male				Female				Male				Female			
	DFS	OS	DMFS	LRRFS	DFS	OS	DMFS	LRRFS	DFS	OS	DMFS	LRRFS	DFS	OS	DMFS	LRRFS
	(%)	(%)	(%)	(%)	(%)	(%)	(%)	(%)	(%)	(%)	(%)	(%)	(%)	(%)	(%)	(%)
A	89.6	93.9	94.6	94.7	91.1	95.6	100	95.3	76.3	83.5	83.4	92.1	72.8	89.4	86.3	85.4
B	88.0	95.9	95.6	92.7	86.0	97.7	93.1	90.4	78.1	83.4	84.9	93.0	74.4	80.4	79.1	88.4
AB	91.8	95.5	91.8	100	100	100	100	100	67.9	78.6	71.4	86.5	88.9	83.3	100	88.9
O	92.6	96.0	95.7	96.3	88.9	96.8	98.4	90.4	70.6	85.3	80.4	86.3	80.8	91.2	89.7	87.6
Non-A	90.8	96.0	95.4	95.2	88.8	97.4	96.6	91.2	73.0	84.2	81.3	88.6	79.0	87.4	86.7	87.9
P1 (A vs. B)	0.677	0.837	0.616	0.632	0.485	0.991	0.081	0.395	0.644	0.976	0.786	0.663	0.629	0.69	0.448	0.564
P2 (A vs. AB)	0.61	0.473	0.622	0.207	0.337	0.503	-	0.493	0.166	0.222	0.103	0.58	0.234	0.867	0.225	0.651
P3 (A vs. O)	0.255	0.123	0.818	0.345	0.708	0.717	0.409	0.332	0.25	0.658	0.456	0.146	0.146	0.397	0.363	0.542
P4 (A vs. non-A)	0.593	0.26	0.773	0.623	0.684	0.762	0.22	0.387	0.447	0.917	0.544	0.371	0.169	0.693	0.706	0.456

Abbreviations: Pre-DNA = pre-treatment Epstein-Barr virus DNA; Pre-L = pre-treatment Epstein-Barr virus DNA < 1355 copies/ml; Pre-H = pre-treatment Epstein-Barr virus DNA ≥ 1355 copies/ml; DFS = disease-free survival; OS = overall survival; DMFS = distant metastasis-free survival; LRRFS = locoregional relapse-free survival

## Discussion

To the best of our knowledge, our study is the first one to clarify the prognostic relationship between plasma EBV DNA and ABO blood group for patients with NPC treated by IMRT. The results of our current study revealed blood group was not an independent prognostic factor in NPC in either the entire cohort or subgroup analysis. The A, B, AB and O blood group distribution of our study was similar to previous studies [11, 12]. Previous studies have resulted in controversy regarding the prognostic difference between A blood group and the non-A groups in NPC [11–13]. Therefore, in this analysis, the clinicopathological features and failure patterns were only compared between A blood and the non-A blood groups to clarify the differences between these groups. Moreover, A blood group was used as the control group, and compared with the individual B, AB, O blood groups as well as the combined non-A blood groups in both univariate and multivariate analysis.

The two studies by Ouyang et al. [11] and Sheng et al. [12] revealed that patients with blood group A had significantly lower DMFS compared to patients with non-A blood groups. Moreover, Zhang et al. [13] showed high VCA-IgA level was associated with a favorable prognosis in male patients with stage II disease who had an A blood type (p = 0.008), compared with those with non-A blood type. The cohorts in these three studies were large and consisted of many basic prognostic factors like tumor stage and host factors. However, the most important prognostic biomarker of plasma EBV DNA was not included in these studies, and it may influence the results after included in the analysis. Our current large cohort study made up for the deficiencies in previous studies, and the results showed that blood group had no prognostic value. A similar non-significant association has also been reported in laryngeal cancer [9]. Two reasonable explanations may account for these non-significant results. Firstly, ABO blood group did not have prognostic value. Secondly, as plasma EBV DNA was not included in the survival analyses of previous studies [11–13], it is possible that any prognostic effect of blood group was diminished by the highly significant prognostic effect of plasma EBV DNA. In the current study, multivariate analysis further confirmed plasma EBV DNA was a powerful prognostic factor for DFS, OS, DMFS and LRRFS (*P* ≤ 0.001 for all rates).

A molecular mechanism underlying the prognostic value of blood group in non-small-cell lung cancer (NSCLC) has been identified. Lee et al. [10] reported that disease progressed significantly earlier in patients with tumors negative for the blood group antigen A than patients with antigen A–positive tumors, indicating that expression of blood group antigen A in tumor cells is a protective prognostic factor in patients with NSCLC. However, it still remains unknown whether this molecular mechanism applies to NPC, as no relevant research has been reported. Therefore, future studies should focus on the mechanism in NPC.

Subgroup analysis of male patients with stage II or III NPC by Zhang et al. [13] revealed a high VCA-IgA level was associated with a better prognosis than low VCA-IgA level in patients with blood group A. Moreover, pre-treatment EBV DNA has been proven to correlate with tumor stage in NPC [23–25]. Therefore, it was reasonable to further investigate the prognostic value of blood group based on different plasma EBV DNA levels. In subgroup analysis by Ouyang et al. [11], the increased risks of OS and DMFS associated with blood group A were only observed in male patients. It [11]was concluded that the negative effects of blood group A in terms of survival in NPC may be confounded by gender which was reported previously[26], since the gender distribution was unbalanced between the patients with blood group A and non-A blood groups. In our current study, the gender distribution was well-balanced, which eliminated this source of bias. However, the results of our subgroup analysis showed that both male and female patients with blood group A had similar prognoses compared to patients with B, AB, O and non-A blood groups. These results may help to further confirm that ABO blood group have no prognostic value in NPC.

Our results indicated that ABO blood group was not an independent prognostic factor after including EBV DNA in the analysis. Therefore, assessment of the prognosis of patients with NPC based on ABO blood group is not relevant to clinical practice. However, the present study is limited by its retrospective nature and potentially insufficient follow-up time, although we selected DFS as the major end-point to address this shortcoming. A longer follow-up time may be necessary in future studies addressing the prognostic value of ABO blood group.

## Conclusion

Based on our current findings, ABO blood group was not identified as an independent factor which could affect the prognosis of patients with NPC treated by IMRT. Moreover, no substantial relationship between ABO blood group and plasma EBV DNA was detected. Therefore, Clinicians may not need to consider ABO blood group when assessing the prognosis of patients with NPC, and this could simplify the clinical treatment strategy.
